# Why do depression, conduct, and hyperactivity symptoms co-occur across adolescence? The role of stable and dynamic genetic and environmental influences

**DOI:** 10.1007/s00787-020-01515-6

**Published:** 2020-04-06

**Authors:** Monika A. Waszczuk, Helena M. S. Zavos, Thalia C. Eley

**Affiliations:** 1 Department of Psychiatry, Stony Brook University, Stony Brook, NY USA; 2 Department of Psychology, Institute of Psychiatry, Psychology and Neuroscience, King’s College London, London, UK; 3 Social, Genetic and Developmental Psychiatry Centre, Institute of Psychiatry, Psychology and Neuroscience, King’s College London, Box PO80, De Crespigny Park, London, SE5 8AF UK

**Keywords:** Adolescence, Comorbidity, Conduct, Depression, Hyperactivity, Twin study

## Abstract

Depression, conduct, and hyperactivity symptoms are chronic and frequently co-occur in adolescence. Common genetic and environmental vulnerability to these conditions have previously been demonstrated, however, the manner in which common versus disorder-specific etiological influences operate across development and maintain symptom co-occurrence is unclear. Thus, the current study investigated the role of common genetic and environmental influences in the comorbidity of depression, conduct, and hyperactivity across adolescence. Over 10,000 twins and their parents reported adolescents’ symptoms at mean ages 11 and 16 years. Biometric independent pathway models were fitted to estimate genetic and environmental contributions to the continuity of symptom co-occurrence over time, as well as time‐ and symptom‐specific influences. Results found that a common stable genetic factor accounted for the concurrent and longitudinal co-occurrence of depression, conduct, and hyperactivity symptoms. New genetic influences common to these three symptom scales emerged at 16 years, and further contributed to symptom co-occurrence. Conversely, environmental influences largely contributed to the time-specific associations. The findings were generally consistent for self- and parent-reported symptoms. Overall, the results suggest that stable, overlapping genetic influences contribute to the co-occurrence of depression, conduct, and hyperactivity symptoms across adolescence. The results are in line with hierarchical causal models of psychopathology, which posit that much of the developmental co-occurrence between different symptoms is due to common liability. Specifically, current findings indicate that only genetic influences constitute common liability over time. Future studies should identify genetically influenced transdiagnostic risk and maintenance factors to inform prevention and treatment of comorbid internalizing and externalizing symptoms in adolescence.

**Electronic supplementary material:**

The online version of this article (10.1007/s00787-020-01515-6) contains supplementary material, which is available to authorized users.

Psychiatric comorbidity, the co-occurrence of disorders at above chance levels, is the rule rather than the exception across development [1]. Disorder and symptom co-occurrence are associated with poorer treatment response, greater impairment, worse illness course, and less optimal long-term outcomes [2–4]. Moreover, psychiatric conditions show homotypic (within-symptom) and heterotypic (across-symptom) continuity over time, with symptoms of one disorder predicting future symptoms of other disorders in a bidirectional manner [5–7]. Given the clinical importance of persistent psychiatric comorbidity, understanding of transdiagnostic risk and maintenance factors common across different symptoms and across development is crucial for informing successful prevention and intervention strategies in young people. Adolescence in particular is a crucial developmental period characterized by biological, social, and psychological transitions that can be impaired by a marked increase in prevalence of depression and externalizing psychopathology [8]. Thus, the current study aimed to understand the role of genetic and environmental influences in the co-occurrence of three common psychiatric symptoms across adolescence: depression, conduct, and hyperactivity.

## Etiology of within-time co-occurrence

Adolescent depression is highly prevalent [9] and commonly co-occurs with externalizing psychopathology, including conduct disorder and ADHD [1, 10, 11]. The co-occurrence of these three disorders and their symptoms is consistent with hierarchical models of psychopathology, which pose a substantial correlation between higher-order internalizing and externalizing factors [7, 12, 13]. Much of this comorbidity is thought to be due to common risk factors, including overlapping genetic and environmental influences [7, 14–16]. For example, both twin and molecular genetic studies find that common genetic influences underpin the within-time co-occurrence of depression, conduct, and hyperactivity symptoms [17–19]. This broad genetic vulnerability to different psychiatric symptoms is in line with the generalist gene hypothesis [20, 21] and with the molecular evidence of a widespread genetic pleiotropy [22, 23]. Likewise, environmental influences also contribute to the co-occurrence of internalizing and externalizing psychopathology, albeit accounting for considerably less covariance than genetic factors [17, 18, 24, 25]. Many studies that have started identifying transdiagnostic environmental risk factors, such as stress, childhood maltreatment, and discrimination [26–31]. Nonetheless, the within-time etiologic overlap between depression, conduct, and hyperactivity symptoms is not absolute, with environmental influences in particular showing considerable symptom specificity [17, 18, 25, 32]. Finally, higher order twin models indicate that etiological overlap is larger for disorders within a domain (e.g. conduct and hyperactivity symptoms, both within externalizing domain) than for disorders across domains (e.g. depression and conduct symptoms) [17, 24, 25].

## Etiology of homotypic continuity

Etiological influences on the homotypic continuity of depression and externalizing symptoms are dynamic across development [33]. In other words, while genes contribute markedly to the within-symptom continuity, there is also evidence for genetic innovation (new influences emerging at later time points) and attenuation (previous influences gradually declining at later time points) over time [34]. Furthermore, while environmental influences tend to be time specific, a small proportion has been found to influence developmental symptom continuity.

## Etiology of heterotypic continuity

Depression and externalizing symptoms co-occur over time [5]. However, the degree to which stable and time-specific etiological influences are shared between depression, conduct, and hyperactivity symptoms over time, and contribute to their co-occurrence across development, remains largely unknown. To date, one study found that in childhood, genetic, but not environmental, influences specific to externalizing symptoms at age 5 years old affect future internalizing symptoms at age 12 [35]. Moreover, another study found that a developmental trajectory characterized by the co-occurrence of emotional and conduct symptoms across ages 4 to 16 was underpinned by moderate genetic influences common to these symptoms [36]. Finally, emerging longitudinal evidence from internalizing symptoms suggests that both genetic and environmental influences underpin co-occurrence of symptoms across adolescence [37–39]. However, to date, the etiology of heterotypic continuity has not been explored for the relationships between internalizing and externalizing psychopathology in this age group. Understanding how genetic and environmental influences contribute to the co-occurrence of internalizing and externalizing symptoms across development might provide clinically relevant insights in the context of growing interest in transdiagnostic interventions, for example by informing research efforts to identify common treatment targets [40–42]. It also directly informs the theoretical models of associations between internalizing and externalizing psychopathology, by estimating the extent of the etiological overlap between these symptoms across adolescence.

## Current study

The aim of the current study was to address gaps in the current understanding of dynamic genetic and environmental influences underpinning the co-occurrence of depression and externalizing symptoms across adolescence. The investigation focuses on depression because it is the single largest contributor to non-fatal health loss globally [43], and its prevalence rates increase markedly in adolescence [9, 44, 45], which is accompanied by changes in etiological influences [33]. Adolescence is also a dynamic developmental period characterized by genetic innovation and attenuation on externalizing psychopathology [33].

Using a large, multiple-informant, epidemiological sample of twins followed prospectively from the age of 11 to 16 years, we investigated the stability and change of genetic and environmental influences shared between depression, conduct, and hyperactivity symptoms. Notably, we elected to study this question using data from both self- and parent-report because there are different strengths and limitations associated with each approach [46, 47], with observers showing only moderate agreement due to their different perspectives [48, 49]. There is also evidence of reporter effects on parameter estimates, e.g. parent report tends to yield higher heritability of ADHD than self-report, although different informants appear to measure a largely common genetic liability [50]. Furthermore, parent-reported symptoms tend to show higher shared-environmental influences than self-report [51].

We hypothesized that: (H1) symptoms of depression, conduct, and hyperactivity would show homotypic and heterotypic continuity across development; (H2) stable, common genetic factors would influence all symptoms from 11 to 16 years, contributing to heterotypic continuity; (H3) environmental influences would be largely time and symptom specific, with relatively smaller contributions to heterotypic continuity; and (H4) genetic and environmental innovation would be observed, a significant proportion of which would contribute to the symptom co-occurrence at 16 years.

## Methods

### Sample

The analyses use data from the Twins Early Development Study (TEDS), an epidemiological study of over 10,000 twin pairs born in England and Wales between 1994 and 1996. Full recruitment details have been reported elsewhere [52, 53]. The current analyses focus on the data collected at two waves when twins were approximately 11 and 16 years old (mean age = 11.23 and 16.32 years, SD = 0.70 and 0.68, respectively), hereon referred to as times 1 and 2 (Table 1). Data collection has been conducted by mailing out questionnaire booklets. The sample is representative of the population in England and Wales in terms of ethnicity and family socioeconomic status [53]. Attrition in the analytic sample from time 1 to time 2 was associated with lower socioeconomic status and higher psychopathology, but the group differences were very small (under quarter SD difference). Informed consent was obtained from parents of all participating adolescents and the study was approved by the Institute of Psychiatry Ethics Committee. Zygosity was established using parent-report questionnaires of physical similarity, which is estimated to be 95% accurate when compared to DNA testing [54]. DNA testing was conducted in cases where zygosity was ambiguous.

**Table 1:** Sample characteristics and descriptive statistics

	Time 1		Time 2	
	Self-report	Parent-report	Self-report	Parent-report
Sample characteristics				
*N* (individual)	11,761	11,760	10,215	10,256
Female (%)	6201 (53%)	6205 (53%)	5665 (55%)	5665 (55%)
MZ (%)	4219 (36%)	4222 (36%)	3625 (36%)	3644 (36%)
MZmale/MZfemale	1909/2310	1904/2318	1498/2127	1516/2128
DZmale/DZfemale/DZoppsex/unknown	1788/2026/3680/48	1792/2022/3678/46	1417/1875/3230/68	1422/1866/3258/66
Descriptive statistics: mean (SD), range, Cronbach’s *α*				
Depression	2.52 (3.62), 0–24, 0.86	1.40 (2.60), 0–24, 0.85	3.27 (4.16), 0–22, 0.88	1.00 (2.31), 0–24, 0.87
Conduct	1.90 (1.66), 0–10, 0.60	1.33 (1.48), 0–10, 0.57	1.65 (1.47), 0–10, 0.54	1.24 (1.40), 0–10, 0.55
Hyperactivity	3.54 (2.31), 0–10, 0.70	2.85 (2.28), 0–10, 0.76	3.57 (2.31), 0–10, 0.73	2.28 (1.99), 0–10, 0.71

Descriptive statistics presented on unregressed and untransformed data for direct comparison with other samples. Participants were excluded if they did not provide consent, if they had severe medical disorders, experienced severe perinatal complications or if their zygosity was unknown (*N* = 316 families)

### Measures

*Depressive symptoms* were measured using the self and parent-report Short Mood and Feelings Questionnaire [55]; a 13-item measure assessing how often depressive symptoms occurred in the past 2 weeks. Due to a content overlap with hyperactivity, question 4 pertaining to symptoms of restlessness was not included in the current analyses. Responses were summed to give total depressive symptom scores. The SMFQ demonstrates very good reliability and validity [55], see Table 1 for internal consistencies in the current sample.

*Conduct and hyperactivity symptoms* were measured using the parent and self-report Strengths and Difficulties Questionnaire, SDQ [56]. The analyses focused on two SDQ subscales consisting of five items each, assessing conduct and hyperactivity behavioral problems. The SDQ is a widely used and validated instrument in adolescents [49]. See Table 1 for internal consistencies in the current sample.

### Analyses

The twin design compares the degree of similarity between monozygotic (MZ, sharing 100% of their genes) and dizygotic (DZ, sharing on average 50% of their segregating genes) twin pairs. These relative differences in within-pair similarities allow calculations of the influences caused by additive genetics (*A*), shared environment (*C*, nongenetic factors that contribute to similarity between twins), and non-shared environment (*E*, nongenetic factors that contribute to differences between twins). Where correlations are higher for MZ pairs than for DZ pairs, it suggests that genetic influences are playing a role in the etiology. Within-pair similarity that is not due to genetic factors is accounted for by *C*, which is evident when DZ correlations are more than half the magnitude of MZ correlations. Finally, within-pair differences between MZ twins inform estimation of *E* influences, and any measurement error present is also included in this term. Presence of gene by environment interaction might result in inflating estimates of *E*, or deflating estimates of *A* [57]. Quantitative genetic designs and methods are described in more detail elsewhere [58].

All analyses were conducted using a structural equation modelling package OpenMx [59] within R (www.R-project.org) [60]. OpenMx has been designed for the analysis of genetically informative data and controls for non-independence of family members. The variables were regressed for age and sex [61] and all variables except hyperactivity were log transformed to correct for skew. All models were fitted using raw data maximum likelihood. The core relative fit statistic was minus twice the log likelihood (− 2LL) of the observations, with differences in − 2LL between models distributed as *χ*^2^, and with lower *χ*^2^ values indicating a better fit. In addition, model fit was assessed using the Akaike’s and the Bayesian’s Information Criterion (AIC and BIC, respectively), with more negative values suggesting a better fit. Moreover, 95% confidence intervals of parameter estimates were obtained by maximum likelihood.

Univariate analyses assessing influences of *A*, *C*, and *E* were conducted on all variables. Sex differences were examined to inform twin modelling. There were scalar (variance) sex differences in all variables except self-report time 1 depression, and time 2 self-report hyperactivity and parent-report conduct. Scalar models were fitted to account for these differences. Furthermore, there were quantitative sex differences in self-report depression at time 2, as previously reported in Waszczuk et al. [62].

**Fig. 1:**
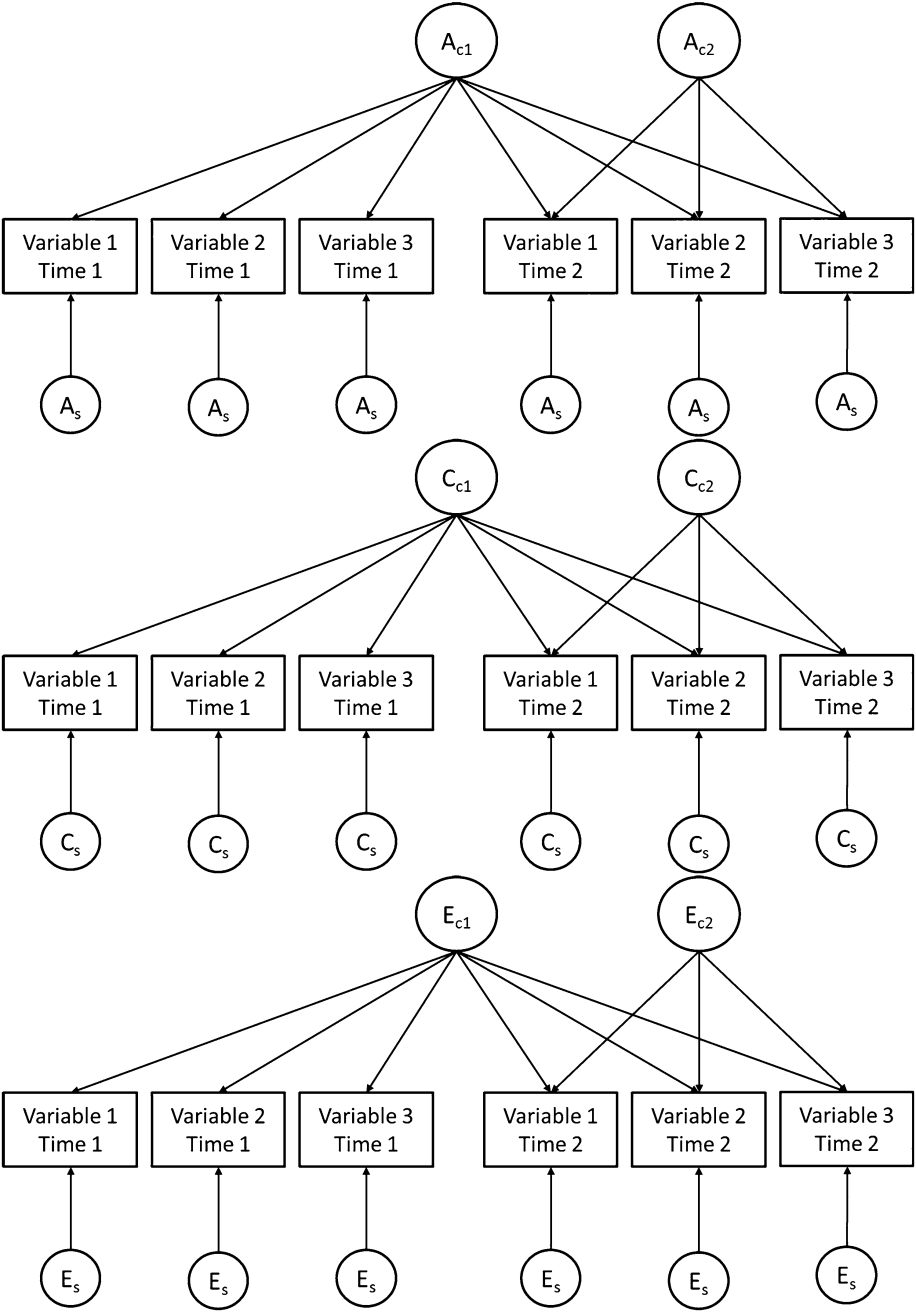
Independent pathway model. *A* additive genetic influences, *C* shared-environmental influences, *E* non-shared environmental influences. Subscript C denotes common influences and subscript S denotes time and variable specific, residual influences

Multivariate analyses were conducted separately for self and parent-report data due to significantly different univariate estimates for corresponding symptoms across raters. Independent pathway models were fitted to assess whether genetic and environmental influences common to the three symptoms at time 1 contribute to the continuity of the symptoms to time 2 (Fig. 1). This was done by fitting common genetic (*A*_C1_), shared (*C*_C1_), and non-shared environmental (*E*_C1_) factors which influenced all variables. The model also estimates common genetic and environmental influences that emerge at time 2, by additionally fitting time 2-specific common genetic (*A*_C2_), shared (*C*_C2_), and non-shared environmental (*E*_C2_) factors loading only on time 2 variables. Moreover, the model estimates symptom- and time-specific residual genetic and environmental influences on each variable at both times (*A*_S_, *C*_S_, and *E*_S_). For completeness, in supplementary analyses, the multivariate model was repeated using measures combined across raters.

## Results

### Phenotypic cross-sectional and longitudinal associations

Descriptive statistics are reported in Table 1. Depression, conduct, and hyperactivity symptoms were moderately associated at both times, with within-time correlations comparable across raters (within-time 1 *r* = 0.36–0.51, within-time 2 = 0.32–0.51, Table 2). Within-symptom (homotypic) continuity of each symptom scale across two time points was moderate, although the stability of externalizing symptoms scales was significantly higher in parent-report (homotypic *r* = 0.26–0.36 in self-report, homotypic *r* = 0.29–0.50 in parent -report). Cross-symptom (heterotypic) continuity was small to moderate and comparable across raters, although co-occurrence of conduct and hyperactivity symptoms across time was somewhat higher in parent-report (heterotypic *r* = 0.16–0.27 in self-report, heterotypic *r* = 0.23–0.39 in parent -report). Notably, the cross-sectional and longitudinal associations between the two externalizing symptoms scales were not much larger in magnitude than their associations with depression.

**Table 2:** Phenotypic correlations. Self-report below diagonal, parent-report above diagonal

	Depression time 1	Conduct time 1	Hyperactivity time 1	Depression time 2	Conduct time 2	Hyperactivity time 2
Depression time 1	1	36 (0.35–0.38)	0.37 (0.35–0.38)	*0.29* (*0.27–0.31*)	**0.26** (**0.24–0.28**)	**0.27** (**0.25–0.29**)
Conduct time 1	0.43 (0.41–0.44)	1	0.46 (0.44–0.47)	**0.24** (**0.21–0.26**)	*0.44* (*0.42–0.46*)	**0.39** (**0.37–0.41**)
Hyperactivity time 1	0.38 (0.37–0.40)	0.51 (0.50–0.53)	1	**0.23** (**0.21–0.25**)	**0.37** (**0.35–0.39**)	*0.50* (*0.48–0.52*)
Depression time 2	*0.26* (*0.24–0.28*)	**0.19** (**0.17–0.22**)	**0.16** (**0.14–0.18**)	1	0.35 (0.33–0.37)	0.32 (0.30–0.34)
Conduct time 2	**0.21** (**0.19–0.23**)	*0.31* (*0.29–0.33*)	**0.25** (**0.23–0.27**)	0.34 (0.32–0.36)	1	0.51 (0.50–0.53)
Hyperactivity time 2	**0.22** (**0.20–0.24**)	**0.27** (**0.25–0.29**)	*0.36* (*0.34–0.38*)	0.35 (0.33–0.37)	0.45 (0.43–0.46)	1

Homotypic (within symptom) continuity highlighted in italics; heterotypic (across symptom) continuity highlighted in bold

### Etiological influences on individual symptom scales

Univariate results for some of the variables have been reported previously [62–64] and are presented in Table S1 in Supplementary Material. In short, depression symptoms were moderately heritable (*A* = 0.32–0.46), while conduct and hyperactivity symptoms were moderately heritable in self-report data (*A* = 0.36–0.45), but highly heritable in parent-report data (*A* = 0.49–0.77). There were small shared-environmental influences on depression and conduct symptoms (*C* = 0.07–0.21, and 0.06–0.28, respectively). Non-shared environmental influences ranged from moderate to high (*E* = 0.22–0.64). Across raters, homotypic continuity of depression was to a comparable degree due to stable genetic and shared environmental influences; homotypic continuity of conduct problems was almost entirely due to stable genetic influences; and homotypic continuity of hyperactivity was due to stable genetic and non-shared environmental influences (Table S2 in Supplementary Material).

### Genetic influences on symptom co-occurrence

**Fig. 2:**
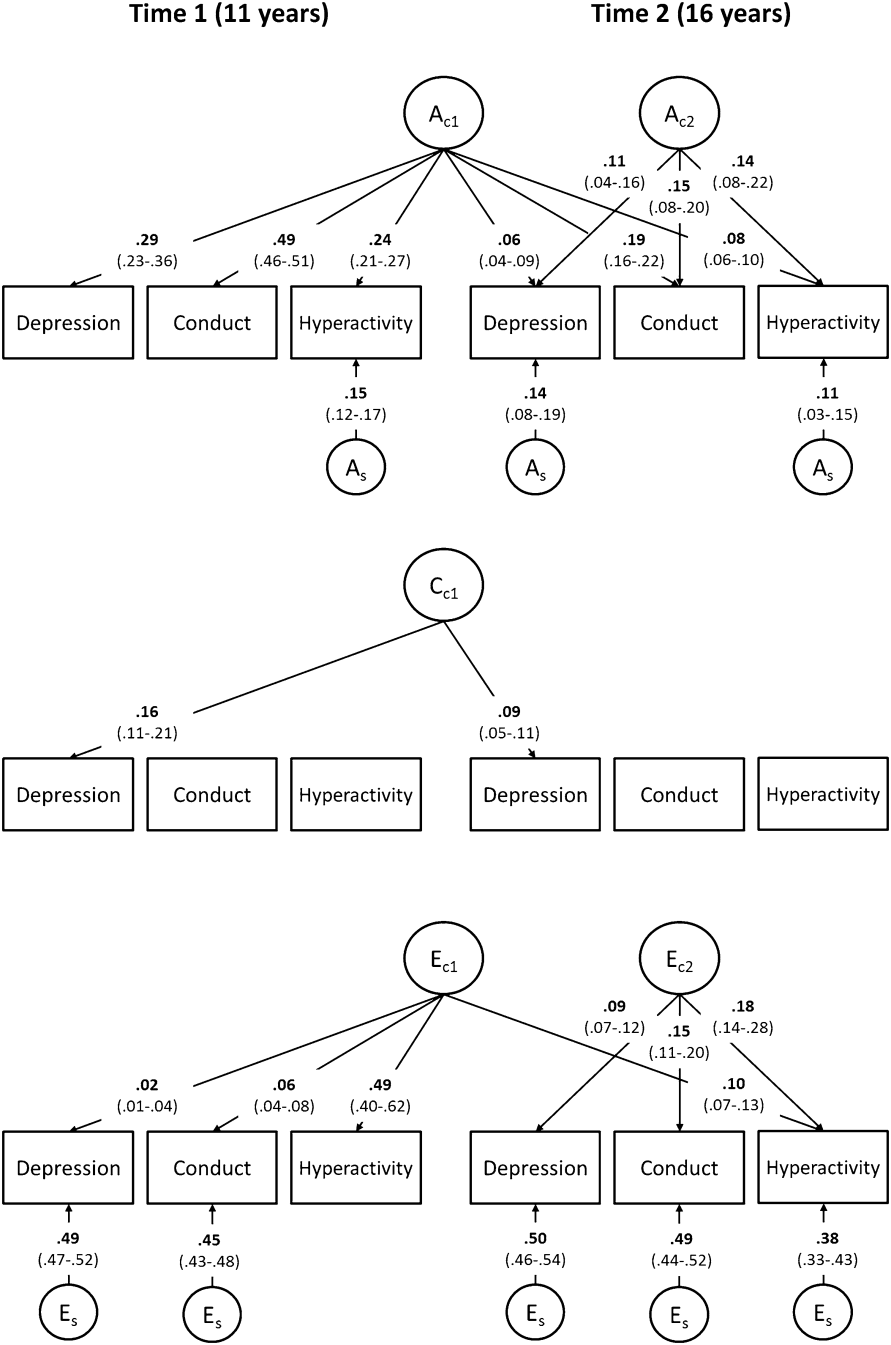
Independent pathways model, significant results for self-report symptoms. *A* additive genetic influences, *C* shared-environmental influences, *E* non-shared environmental influences. Subscript C denotes common influences and subscript S denotes time and variable specific, residual influences. For example, *A*_C1_ shows the first set of common additive genetic influences that load significantly on all symptoms at times 1 and 2, contributing to the co-occurrence of symptoms over time. *A*_C2_ is the second set of common additive genetic influences that load significantly on all symptoms at time 2, indicating transdiagnostic genetic influences that emerged at 16 years, further contributing to the symptom co-occurrence at time 2. Furthermore, there are residual *A*_S_ on hyperactivity at times 1 and 2, and depression at time 2, indicating symptom-specific additive genetic etiology of these problems. Full model presented in Fig. 1 was fitted, but only results for significant paths are shown. Non-significant paths were not dropped to prevent artificially inflating remaining paths. All paths presented are squared for standardization: *A*, *C*, and *E* influences on each variable (including non-significant paths that are not shown) add up to 1. Square root of these values should be taken to obtain variance path. For full results including non-significant paths, see Table S3 in the Supplementary Material

**Fig. 3:**
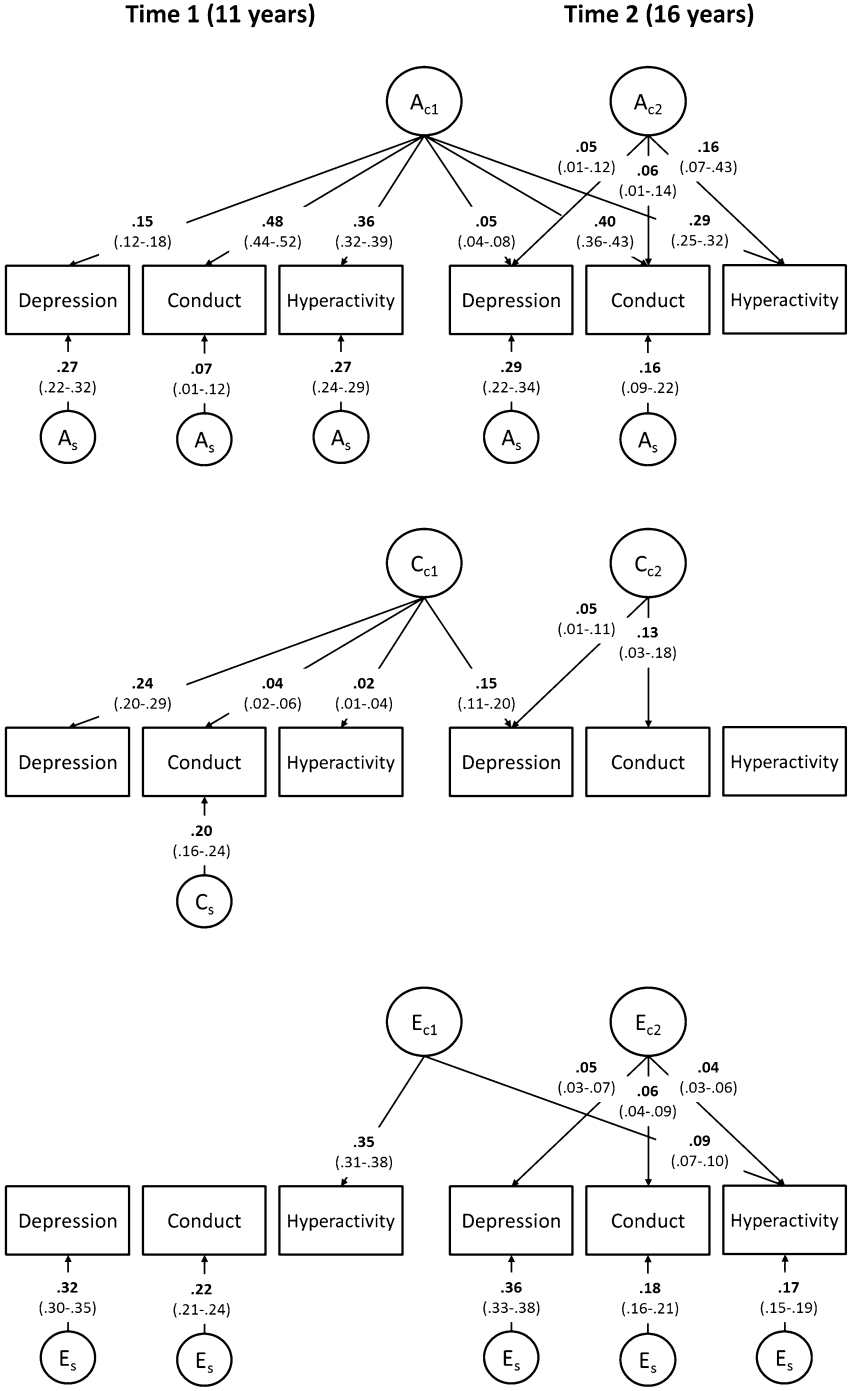
Independent pathway model, significant results for parent-report symptoms. *A* additive genetic influences, *C* shared-environmental influences, *E* non-shared environmental influences. Subscript C denotes common influences and subscript S denotes time and variable specific, residual influences. For example, *A*_C1_ shows the first set of common additive genetic influences that load significantly on all symptoms at times 1 and 2, contributing to the co-occurrence of symptoms over time. *A*_C2_ is the second set of common additive genetic influences that load significantly on all symptoms at time 2, indicating transdiagnostic genetic influences that emerged at 16 years, further contributing to the symptom co-occurrence at time 2. Furthermore, there are significant residual *A*_S_ on all symptoms except hyperactivity time 2, indicating considerable additive genetic etiology specific to symptom and developmental stage. Full model presented in Fig. 1 was fitted, but only results for significant paths are shown. Non-significant paths were not dropped to prevent artificially inflating remaining paths. All paths presented are squared for standardization: *A*, *C*, and *E* influences on each variable (including non-significant paths that are not shown) add up to 1. Square root of these values should be taken to obtain variance path. For full results including non-significant paths, see Table S4 in the Supplementary Material

Genetic influences accounted for the largest proportion of the bivariate phenotypic correlations between the three symptoms (*h*^2^ of phenotypic correlations = 0.56 to 1.00, Table S2 in Supplementary Material). Accordingly, the independent pathway models in both self- and parent-report data found significant genetic influences common to depression, conduct, and hyperactivity symptoms operating across both time points (*A*_C1_ = 0.06–0.49 in self-report, *A*_C1_ = 0.05–0.48 in parent-report data, Figs. 2 and 3, respectively). This indicates that genetic influences shared by these three symptoms at time 1 continue to influence depression, conduct, and hyperactivity symptoms at time 2, contributing to both homotypic and heterotypic continuity. A second set of genetic influences common to time 2 symptoms was also significant (*A*_C2_ = 0.11–0.14 in self-report, *A*_C1_ = 0.05–0.16 in parent-report data), indicating that new genetic influences emerged at time 2, and broadly influenced depression and two externalizing symptoms, further contributing to their co-occurrence at time 2. Finally, some genetic influences were specific to only one symptom scale, contributing to change in symptoms over time. Parent-report model was characterized by more of such time- and symptom-specific residual genetic influences than the self-report model (*A*_S_ = 0.11–0.15 in self-report, *A*_*S*_ = 0.07–0.29 in parent-report data). Overall, genetic influences operated largely in a transdiagnostic manner and contributed to the symptom co-occurrence at each time point, as well as across time. Finally, a very similar pattern of results emerged when the model was fitted using measures combined across self- and parent-rating, see Fig. S1 in the Supplementary Material.

### Shared-environmental influences on symptom co-occurrence

The common shared-environmental factor influenced only depression at times 1 and 2 in self-report data (*C*_C1_ = 0.16 and 0.09, respectively, Fig. 2), thus it only contributed to the homotypic continuity of depression. In the parent-report model, shared-environmental factors were more pronounced: the common shared-environmental factor most strongly influenced depression at times 1 and 2 (*C*_C1_ = 0.24 and 0.15, respectively, Fig. 3), but in addition showed small but significant influences on conduct and hyperactivity at time 1 (*C*_C1_ = 0.02–0.04). Moreover, in the parent-report data, there was a second common shared-environmental factor influencing depression and conduct at time 2 (*C*_C2_ = 0.05 and 0.13, respectively). Thus, shared-environmental influences operated in a transdiagnostic manner at each time point in parent-report data, but did not contribute to co-occurrence of symptoms across the two time points.

### Non-shared environmental influences on symptom co-occurrence

In self-report data, the common non-shared environmental factor influenced largely hyperactivity at times 1 and 2 (*E*_C1_ = 0.49 and 0.10, respectively, Fig. 2), contributing to homotypic continuity of hyperactivity symptoms, with small significant influences on time 1 depression and conduct (*E*_C1_ = 0.02 and 0.06, respectively). Similarly, in parent-report data, the common non-shared environmental factor influenced only hyperactivity at times 1 and 2 (*E*_C1_ = 0.35 and 0.09, respectively, Fig. 3), contributing to the homotypic continuity of hyperactivity symptoms. The common non-shared environmental influences specific to time 2 loaded on all three symptoms in both models (*E*_C2_ = 0.09–0.18 in self-report data, *E*_C2_ = 0.04–0.06 in parent-report data), indicating common environmental etiology of depression, conduct, and hyperactivity at time 2. In both self- and parent-report models, residual non-shared environmental influences were significant on all symptoms (*E*_S_ = 0.38–0.50 in self-report data, *E*_S_ = 0.17–0.36 in parent-report data), except time 1 hyperactivity. In sum, non-shared environmental influences were largely symptom and time specific, and contributed to change in symptoms over time. Some non-shared environmental factors contributed to symptom co-occurrence cross-sectionally, but did not contribute to the symptom co-occurrence over time.

## Discussion

The current study investigated how common etiological influences contribute to the co-occurrence of depression, conduct, and hyperactivity symptoms across adolescence. The results indicated homotypic and heterotypic continuity of these symptoms, which were largely underpinned by stable, transdiagnostic genetic influences. These findings, which were remarkably similar for self- and parent-report symptoms, are in line with hierarchical causal models of psychopathology, which suggest that much of the developmental co-occurrence between different symptoms is due to common liability. Specifically, the current findings indicate that only genetic influences constitute common liability over time, whereas common environmental influences were time specific. Thus, genetically influenced transdiagnostic risk factors may account for the longitudinal co-occurrence of depression, conduct, and hyperactivity symptoms across adolescence.

The moderate heterotypic associations between depression, conduct, and hyperactivity symptoms across adolescence observed in the current study are in line with previous work demonstrating comorbidity and heterotypic continuity between these symptoms [1, 5, 10, 11]. Notably, conduct and hyperactivity were comparably associated with each other as they were with depression symptoms, in contrast with previous studies in adults reporting much higher continuity within than across internalizing and externalizing domains [5].

Going beyond previous findings, the current study is the first to show that this pattern of longitudinal co-occurrence is in part explained by a stable, common genetic factor influencing all symptoms. Moreover, there was evidence for genetic innovation that further contributed to the comorbidity, with a second set of genetic influences common to all symptoms coming online at 16 years. Thus, the current study provides preliminary evidence that both stable and time-specific genetic influences have transdiagnostic effects (i.e. on both depression and externalizing symptoms), contributing to the enduring high genetic overlap between symptoms over time. The results are also in line with previous findings of common genetic influences explaining the links between internalizing and externalizing psychopathology across childhood [35], but extend these results to adolescence. Nonetheless, unlike Wertz et al. [35], in our older age group, we did not find that genetic influences specific to externalizing psychopathology contribute to future internalizing symptoms. Finally, the results compliment recent findings by Hannigan et al. [36] that a developmental trajectory characterized by the co-occurrence of emotional and conduct symptoms is heritable, but extend these findings to include hyperactivity symptoms and explicate the role of genetic innovation in maintaining this association.

While environmental influences contributed modestly to some of the within-time associations among depression, conduct, and hyperactivity symptoms, these common environmental influences were time specific and did not contribute to the symptom co-occurrence over time. Instead, we found that shared and non-shared environmental influences contributed only to the homotypic continuity of depression and hyperactivity, respectively, in line with previous findings from early to middle adolescence [65, 66]. Most notably, majority of non-shared environmental influences were time and symptom specific, contributing to symptom discontinuity over time. Twin studies cannot provide information about which environmental influences contributed to within-time symptom co-occurrence without directly measuring exposures and experiences, but it is plausible that these could constitute transient factors such as episodic stressful life events, e.g. accidents or conflicts with peers [67, 68]. Conversely, some of the long-lasting environmental exposures that maintain depression and hyperactivity symptoms across adolescence could constitute chronic stressors and sociocultural influences, for example a family environment or socio-economic status [68–70]. While the effects of stress and trauma are known to be transdiagnostic [27], the current study tentatively suggests that such environmental effects may not contribute beyond cross-sectional co-occurrence, to influence heterotypic continuity across depression, conduct, and hyperactivity. Future twin studies should include measures of environmental risk factors and identify which of them operate in episodic vs. chronic manner to inform precise intervention targets.

Both phenotypic and genetic results support hierarchical causal models of psychopathology in explaining the association between depression, conduct, and hyperactivity symptoms over time [7, 14]. Future studies should identify transdiagnostic genetic risk factors, including polygenic risk scores, implicated in comorbidity, to inform prediction, prevention, and treatment approaches [23, 71, 72]. For example, such genetic tools would explicitly capture pleiotropic genetic effects, which in the future might help predict individual’s vulnerability to a broad range of co-occurring and chronic psychopathology, or help identify a subgroup of individuals at the highest genetic risk for recurrent, cross-disorder psychiatric illness course. Moreover, the current results suggests that it might be possible to identify common, genetically influenced downstream vulnerability factors that cut across diagnostic boundaries and maintain these three co-occurring conditions across development. For example, negative emotionality (neuroticism) might be one common feature underlying heterotypic continuity of internalizing and externalizing psychopathology [15].

Simultaneously, the current results suggest that the genetic overlap is not absolute and there are significant genetic influences specific to each symptom. This supports molecular genetic approaches that focus on narrow psychiatric definitions to reduce heterogeneity [73]. Genetic tools such as polygenic risk scores derived for homogenous phenotypes might in the future benefit from a greater precision in predicting specific psychiatric outcomes. Moreover, significant residual genetic influences suggest that symptom-specific, genetically influenced downstream vulnerability factors should continue to be identified alongside transdiagnostic risk factors. One such heritable risk factor specific to externalizing psychopathology might be daring (novelty seeking) personality trait [74].

Finally, the overall pattern of results emerged in self- and parent-report data, providing a multi-informant validation of the heterotypic associations and etiological influences. Nonetheless, there were some notable rater differences. Specifically, according to parent ratings, continuity of externalizing symptoms across adolescence was higher, and transdiagnostic shared-environmental influences were more pronounced, contributing to the within-time symptom overlap. These discrepancies might be due to different perspectives between parents and adolescents [48, 49], and are in line with previous studies finding that parent-reported symptoms tend to show higher shared-environmental influences than self-report [51]. Despite these informant differences, the current pattern of results appears to be robust and replicates across raters, as well as when measures were combined across raters.

### Limitations

The large, genetically informative, longitudinal, and multi-informant sample is the strength of the study. However, a number of limitations are worth noting. First, the conduct problem subscales, although measured using the well-validated SDQ widely used in clinical practice and epidemiology, yielded low internal consistency scores. Low internal consistency for this subscale is not specific to this study [49] and could have increased measurement error and consequently underestimate associations with other symptoms. Nonetheless, the advantages afforded by a broad, non-redundant content coverage, and a quickly administrable instrument with few items per scale feasible for large-scale data collection outweigh the limitation of modest Cronbach’s *α* values [75]. Second, although our results were similar across informant symptom ratings, the results may not be generalizable to diagnosed disorders. Symptom-based approach was taken because symptoms are important markers of psychopathology [76, 77], quantitative phenotypes better capture illness severity and characterize subthreshold cases than categorical diagnoses [78, 79], and common mental disorders are considered to be the extremes of quantitative traits underpinned by the same genetic liability [80, 81].

Third, we did not formally test rater bias, such as a potential inflation of shared environmental influences in parent-report models. Nonetheless, with the exception of conduct problems, shared environmental estimates were not higher in parent models than self-report models, and results were comparable for measures combined across raters. Fourth, we did not measure other internalizing and externalizing symptoms, such as anxiety, and future research should extend our findings to a wider range of psychiatric symptoms, ideally to study the etiological influences on the developmental continuity of the broad higher order psychopathology factors [31, 82]. While we focused on the best measures of the three constructs available, the use of different instruments to assess conduct and hyperactivity vs. depression symptoms could has impacted the relative magnitude of estimates. Finally, there are a number of limitations inherent to the twin design, comprehensively discussed elsewhere [58]. These limitations have minimal and contrasting effects but suggest that parameter estimates should be taken as indicative rather than absolute values.

## Conclusions

The current study investigated how genetic and environmental influences contribute to the co-occurrence of depression, conduct, and hyperactivity symptoms across adolescence. The results indicated bidirectional associations between these three symptoms, which were underpinned by stable, transdiagnostic genetic influences. New genetic influences common to three symptoms emerged at 16 years, and further contributed to symptoms co-occurrence. Taken together, these results are in line with hierarchical causal models of psychopathology, and point to shared, genetically driven mechanisms that contribute to the comorbidity between depression and externalizing symptoms across adolescence.

## Electronic supplementary material

Below is the link to the electronic supplementary material.

Supplementary file1 (DOCX 173 kb)

## References

[1] Angold A, Costello EJ, Erkanli A (1999). Comorbidity. J Child Psychol Psychiatry.

[2] Hudson JL, Keers R, Roberts S, Coleman JR, Breen G, Arendt K, Bögels S, Cooper P, Creswell C, Hartman C (2015). Clinical predictors of response to cognitive-behavioral therapy in pediatric anxiety disorders: the Genes for Treatment (GxT) study. J Am Acad Child Adolesc Psychiatry.

[3] Connor DF, Steeber J, McBurnett K (2010). A review of attention-deficit/hyperactivity disorder complicated by symptoms of oppositional defiant disorder or conduct disorder. J Dev Behav Pediatr.

[4] Lewinsohn PM, Rohde P, Seeley JR (1995). Adolescent psychopathology: III. The clinical consequences of comorbidity. J Am Acad Child Adolesc Psychiatry.

[5] Lahey BB, Zald DH, Hakes JK, Krueger RF, Rathouz PJ (2014). Patterns of heterotypic continuity associated with the cross-sectional correlational structure of prevalent mental disorders in adults. JAMA Psychiatry.

[6] Gregory AM, Caspi A, Moffitt TE, Koenen K, Eley TC, Poulton R (2007). Juvenile mental health histories of adults with anxiety disorders. Am J Psychiatry.

[7] Lahey BB, Krueger RF, Rathouz PJ, Waldman ID, Zald DH (2016). A hierarchical causal taxonomy of psychopathology across the life span. Psychol Bull.

[8] Cicchetti D, Rogosch FA (2002). A developmental psychopathology perspective on adolescence. J Consult Clin Psychol.

[9] Costello EJ, Mustillo S, Erkanli A, Keeler G, Angold A (2003). Prevalence and development of psychiatric disorders in childhood and adolescence. Arch Gen Psychiatry.

[10] Daviss WB (2008). A review of co-morbid depression in pediatric ADHD: etiologies, phenomenology, and treatment. J Child Adolesc Psychopharmacol.

[11] Wolff JC, Ollendick TH (2006). The comorbidity of conduct problems and depression in childhood and adolescence. Clin Child Fam Psychol Rev.

[12] Krueger RF, Kotov R, Watson D, Forbes MK, Eaton NR, Ruggero CJ, Simms LJ, Widiger TA, Achenbach TM, Bach B (2018). Progress in achieving quantitative classification of psychopathology. World Psychiatry.

[13] Kotov R, Krueger RF, Watson D, Achenbach TM, Althoff RR, Bagby M, Brown TA, Carpenter WT, Caspi A, Clark LA, Eaton NR, Forbes MK, Forbush KT, Goldberg D, Hasin D, Hyman SE, Ivanova MY, Lynam DR, Markon K, Miller JD, Moffitt TE, Morey LC, Ormel J, Patrick CJ, Regier DA, Rescorla L, Robinson E, Ruggero CJ, Samuel DB, Sellbom M, Simms LJ, Skodol AE, Slade T, South SC, Tackett JL, Waldman ID, Waszczuk MA, Widiger TA, Wright AGC, Zimmerman M (2017). The Hierarchical Taxonomy Of Psychopathology (HiTOP): a dimensional alternative to traditional nosologies. J Abnorm Psychol.

[14] Krueger RF, Markon KE (2006). Reinterpreting comorbidity: a model-based approach to understanding and classifying psychopathology. Annu Rev Clin Psychol.

[15] Rhee SH, Lahey BB, Waldman ID (2015). Comorbidity among dimensions of childhood psychopathology: converging evidence from behavior genetics. Child Dev Perspect.

[16] Waszczuk MA, Eaton NR, Krueger RF, Shackman AJ, Waldman ID, Zald DH, Lahey BB, Patrick CJ, Conway C, Ormel J, Hyman SE, Fried EI, Forbes MK, Althoff RR, Bach B, Chmielewski M, DeYoung CG, Docherty AR, Forbush KT, Hallquist M, Hopwood CJ, Ivanova MY, Jonas KG, Latzman RD, Markon K, Mullins-Sweatt SN, Pincus AL, Reininghaus U, South SC, Tackett JL, Watson D, Wright AGC, Kotov R (2020). Redefining phenotypes to advance psychiatric genetics: implications from hierarchical taxonomy of psychopathology. J Abnorm Psychol.

[17] Cosgrove VE, Rhee SH, Gelhorn HL, Boeldt D, Corley RC, Ehringer MA, Young SE, Hewitt JK (2011). Structure and etiology of co-occurring internalizing and externalizing disorders in adolescents. J Abnorm Child Psychol.

[18] Rowe R, Rijsdijk FV, Maughan B, Hosang GM, Eley TC (2008). Heterogeneity in antisocial behaviours and comorbidity with depressed mood: a behavioural genetic approach. J Child Psychol Psychiatry.

[19] Anttila V, Bulik-Sullivan B, Finucane HK, Walters RK, Bras J, Duncan L, Escott-Price V, Falcone GJ, Gormley P, Malik R (2018). Analysis of shared heritability in common disorders of the brain. Science.

[20] Eley TC (1997). General genes: a new theme in developmental psychopathology. Curr Dir Psychol Sci.

[21] Plomin R, Kovas Y (2005). Generalist genes and learning disabilities. Psychol Bull.

[22] Gizer IR (2016). Molecular genetic approaches to understanding the comorbidity of psychiatric disorders. Dev Psychopathol.

[23] Smoller JW, Andreassen OA, Edenberg HJ, Faraone SV, Glatt SJ, Kendler KS (2018). Psychiatric genetics and the structure of psychopathology. Mol Psychiatry.

[24] Mikolajewski AJ, Allan NP, Hart SA, Lonigan CJ, Taylor J (2013). Negative affect shares genetic and environmental influences with symptoms of childhood internalizing and externalizing disorders. J Abnorm Child Psychol.

[25] Lahey BB, Van Hulle CA, Singh AL, Waldman ID, Rathouz PJ (2011). Higher-order genetic and environmental structure of prevalent forms of child and adolescent psychopathology. Arch Gen Psychiatry.

[26] Kendler KS, Eaves LJ, Loken EK, Pedersen NL, Middeldorp CM, Reynolds C, Boomsma D, Lichtenstein P, Silberg J, Gardner CO (2011). The impact of environmental experiences on symptoms of anxiety and depression across the life span. Psychol Sci.

[27] Vachon DD, Krueger RF, Rogosch FA, Cicchetti D (2015). Assessment of the harmful psychiatric and behavioral effects of different forms of child maltreatment. JAMA Psychiatry.

[28] Anda RF, Felitti VJ, Bremner JD, Walker JD, Whitfield C, Perry BD, Dube SR, Giles WH (2006). The enduring effects of abuse and related adverse experiences in childhood. Eur Arch Psychiatry Clin Neurosci.

[29] Keyes KM, Eaton NR, Krueger RF, McLaughlin KA, Wall MM, Grant BF, Hasin DS (2012). Childhood maltreatment and the structure of common psychiatric disorders. Br J Psychiatry.

[30] Eaton NR (2014). Transdiagnostic psychopathology factors and sexual minority mental health: evidence of disparities and associations with minority stressors. Psychol Sex Orientat Gend Divers.

[31] Lahey BB, Applegate B, Hakes JK, Zald DH, Hariri AR, Rathouz PJ (2012). Is there a general factor of prevalent psychopathology during adulthood?. J Abnorm Psychol.

[32] Spatola CA, Fagnani C, Pesenti-Gritti P, Ogliari A, Stazi MA, Battaglia M (2007). A general population twin study of the CBCL/6-18 DSM-oriented scales. J Am Acad Child Adolesc Psychiatry.

[33] Hannigan LJ, Walaker N, Waszczuk MA, McAdams TA, Eley TC (2017). Aetiological influences on stability and change in emotional and behavioural problems across development: a systematic review. Psychopathol Rev.

[34] Kendler KS, Gardner CO, Lichtenstein P (2008). A developmental twin study of symptoms of anxiety and depression: evidence for genetic innovation and attenuation. Psychol Med.

[35] Wertz J, Zavos H, Matthews T, Harvey K, Hunt A, Pariante CM, Arseneault L (2014). Why some children with externalising problems develop internalising symptoms: testing two pathways in a genetically sensitive cohort study. J Child Psychol Psychiatry.

[36] Hannigan LJ, Pingault J-B, Krapohl E, McAdams TA, Rijsdijk FV, Eley TC (2018). Genetics of co-developing conduct and emotional problems during childhood and adolescence. Nat Hum Behav.

[37] Waszczuk MA, Zavos HMS, Gregory AM, Eley TC (2016). The stability and change of etiological influences on depression, anxiety symptoms and their co-occurrence across adolescence and young adulthood. Psychol Med.

[38] Silberg JL, Bulik CM (2005). The developmental association between eating disorders symptoms and symptoms of depression and anxiety in juvenile twin girls. J Child Psychol Psychiatry.

[39] Waszczuk MA, Waaktaar T, Eley TC, Torgersen S (2019). Aetiological influences on continuity and co-occurrence of eating disorders symptoms across adolescence and emerging adulthood. Int J Eat Disord.

[40] Conway CC, Forbes MK, Forbush KT, Fried EI, Hallquist MN, Kotov R, Mullins-Sweatt SN, Shackman AJ, Skodol AE, South SC (2018). A hierarchical taxonomy of psychopathology can transform mental health research. Perspect Psychol Sci.

[41] Barlow DH, Farchione TJ, Bullis JR, Gallagher MW, Murray-Latin H, Sauer-Zavala S, Bentley KH, Thompson-Hollands J, Conklin LR, Boswell JF (2017). The unified protocol for transdiagnostic treatment of emotional disorders compared with diagnosis-specific protocols for anxiety disorders: a randomized clinical trial. JAMA Psychiatry.

[42] Marchette LK, Weisz JR (2017). Practitioner Review: empirical evolution of youth psychotherapy toward transdiagnostic approaches. J Child Psychol Psychiatry.

[43] World Health Organization (2017). Depression and other common mental disorders: global health estimates.

[44] Ford T, Goodman R, Meltzer H (2003). The British Child and Adolescent Mental Health Survey 1999: the prevalence of DSM-IV disorders. J Am Acad Child Adolesc Psychiatry.

[45] Moffitt TE, Harrington H, Caspi A, Kim-Cohen J, Goldberg D, Gregory AM, Poulton R (2007). Depression and generalized anxiety disorder: cumulative and sequential comorbidity in a birth cohort followed prospectively to age 32 years. Arch Gen Psychiatry.

[46] Jensen PS, Rubio-Stipec M, Canino G, Bird HR, Dulcan MK, Schwab-Stone ME, Lahey BB (1999). Parent and child contributions to diagnosis of mental disorder: are both informants always necessary?. J Am Acad Child Adolesc Psychiatry.

[47] Bird HR, Gould MS, Staghezza B (1992). Aggregating data from multiple informants in child psychiatry epidemiological research. J Am Acad Child Adolesc Psychiatry.

[48] Stanger C, Lewis M (1993). Agreement among parents, teachers, and children on internalizing and externalizing behavior problems. J Clin Child Psychol.

[49] Goodman R (2001). Psychometric properties of the strengths and difficulties questionnaire. J Am Acad Child Adolesc Psychiatry.

[50] Merwood A, Greven C, Price T, Rijsdijk F, Kuntsi J, McLoughlin G, Larsson H, Asherson P (2013). Different heritabilities but shared etiological influences for parent, teacher and self-ratings of ADHD symptoms: an adolescent twin study. Psychol Med.

[51] Burt SA (2009). Rethinking environmental contributions to child and adolescent psychopathology: a meta-analysis of shared environmental influences. Psychol Bull.

[52] Haworth CMA, Davis OSP, Plomin R (2013). Twins Early Development Study (TEDS): a genetically sensitive investigation of cognitive and behavioral development from childhood to young adulthood. Twin Res Hum Genet.

[53] Rimfeld K, Malanchini M, Spargo T, Spickernell G, Selzam S, McMillan A, Dale P, Eley TC, Plomin R (2019). Twins Early Development Study: a genetically sensitive investigation into behavioural and cognitive development from infancy to emerging adulthood. Twin Res Hum Genet.

[54] Price TS, Freeman B, Craig IW, Petrill SA, Ebersole L, Plomin R (2000). Infant zygosity can be assigned by parental report questionnaire data. Twin Res.

[55] Angold A, Costello EJ, Messer SC, Pickles A, Winder F, Silver D (1995). The development of a short questionnaire for use in epidemiological studies of depression in children and adolescents. Int J Methods Psychiatr Res.

[56] Goodman R (1997). The Strengths and Difficulties Questionnaire: a research note. J Child Psychol Psychiatry.

[57] Rijsdijk FV, Sham PC (2002). Analytic approaches to twin data using structural equation models. Brief Bioinform.

[58] Plomin R, DeFries JC, Knopik VS, Neiderhiser JM (2013). Behavioral genetics.

[59] Boker S, Neale M, Maes H, Wilde M, Spiegel M, Brick T, Spies J, Estabrook R, Kenny S, Bates T (2011). OpenMx: an open source extended structural equation modeling framework. Psychometrika.

[60] TeamRDC (2010). R: a language and environment for statistical computing.

[61] McGue M, Bouchard TJ (1984). Adjustment of twin data for the effects of age and sex. Behav Genet.

[62] Waszczuk MA, Zavos HMS, Antonova E, Haworth CMA, Plomin R, Eley TC (2015). A multivariate twin study of trait mindfulness, depressive symptoms and anxiety sensitivity. Depress Anxiety.

[63] Pingault J-B, Rijsdijk F, Zheng Y, Plomin R, Viding E (2015). Developmentally dynamic genome: evidence of genetic influences on increases and decreases in conduct problems from early childhood to adolescence. Sci Rep.

[64] Lewis GJ, Haworth C, Plomin R (2014). Identical genetic influences underpin behavior problems in adolescence and basic traits of personality. J Child Psychol Psychiatry.

[65] Scourfield J, Rice F, Thapar A, Harold GT, Martin N, McGuffin P (2003). Depressive symptoms in children and adolescents: changing aetiological influences with development. J Child Psychol Psychiatry Allied Discipl.

[66] Bezdjian S, Tuvblad C, Wang P, Raine A, Baker LA (2014). Motor impulsivity during childhood and adolescence: a longitudinal biometric analysis of the go/no-go task in 9- to 18-year-old twins. Dev Psychol.

[67] Hammen C (2005). Stress and depression. Annu Rev Clin Psychol.

[68] March-Llanes J, Marqués-Feixa L, Mezquita L, Fañanás L, Moya-Higueras J (2017). Stressful life events during adolescence and risk for externalizing and internalizing psychopathology: a meta-analysis. Eur Child Adolesc Psychiatry.

[69] Sanchez YM, Lambert SF, Cooley-Strickland M (2013). Adverse life events, coping and internalizing and externalizing behaviors in urban African American youth. J Child Fam Stud.

[70] Kim KJ, Conger RD, Elder GH, Lorenz FO (2003). Reciprocal influences between stressful life events and adolescent internalizing and externalizing problems. Child Dev.

[71] Selzam S, Coleman JR, Caspi A, Moffitt TE, Plomin R (2018). A polygenic p factor for major psychiatric disorders. Transl Psychiatry.

[72] Allegrini AG, Cheesman R, Rimfeld K, Selzam S, Pingault J-B, Eley T, Plomin R (2019) The p factor: genetic analyses support a general dimension of psychopathology in childhood and adolescence. bioRxiv:591354 10.1111/jcpp.13113 PMC6906245 31541466

[73] Hodgson K, McGuffin P, Lewis CM (2017). Advancing psychiatric genetics through dissecting heterogeneity. Hum Mol Genet.

[74] Tackett JL, Waldman ID, Van Hulle CA, Lahey BB (2011). Shared genetic influences on negative emotionality and major depression/conduct disorder comorbidity. J Am Acad Child Adolesc Psychiatry.

[75] Clark LA, Watson D (1995). Constructing validity: basic issues in objective scale development. Psychol Assess.

[76] Fergusson DM, Horwood LJ, Ridder EM, Beautrais AL (2005). Subthreshold depression in adolescence and mental health outcomes in adulthood. Arch Gen Psychiatry.

[77] Balázs J, Miklósi M, Keresztény Á, Hoven CW, Carli V, Wasserman C, Apter A, Bobes J, Brunner R, Cosman D (2013). Adolescent subthreshold-depression and anxiety: psychopathology, functional impairment and increased suicide risk. J Child Psychol Psychiatry.

[78] Markon KE, Chmielewski M, Miller CJ (2011). The reliability and validity of discrete and continuous measures of psychopathology: a quantitative review. Psychol Bull.

[79] Shea MT, Stout R, Gunderson J, Morey LC, Grilo CM, McGlashan T, Skodol AE, Dolan-Sewell R, Dyck I, Zanarini MC (2002). Short-term diagnostic stability of schizotypal, borderline, avoidant, and obsessive-compulsive personality disorders. Am J Psychiatry.

[80] Plomin R, Haworth CM, Davis OS (2009). Common disorders are quantitative traits. Nat Rev Genet.

[81] Martin J, Taylor MJ, Lichtenstein P (2017). Assessing the evidence for shared genetic risks across psychiatric disorders and traits. Psychol Med.

[82] Caspi A, Houts RM, Belsky DW, Goldman-Mellor SJ, Harrington H, Israel S, Meier MH, Ramrakha S, Shalev I, Poulton R (2014). The p factor one general psychopathology factor in the structure of psychiatric disorders?. Clin Psychol Sci.

