# Supplementary material for: Why do depression, conduct, and hyperactivity symptoms co-occur across adolescence? The role of stable and dynamic genetic and environmental influences
Source: Eur Child Adolesc Psychiatry. 2020 Apr 6;30(7):1013–25. doi: 10.1007/s00787-020-01515-6 (PMC8295149; doi:10.1007/s00787-020-01515-6)
Supplement: Supplementary file 1 — Supplementary file1 (DOCX 173 kb) [file 787_2020_1515_MOESM1_ESM.docx]

**SUPPLEMENTARY MATERIALS**

Waszczuk, M. A., Zavos, H. M. S., & Eley T. C. Why do depression, conduct, and hyperactivity symptoms co-occur across adolescence? The role of stable and dynamic genetic and environmental influences.

**Table S1** – Univariate genetic and environmental influences

|  | **A** | **C** | **E** |
| --- | --- | --- | --- |
| **Self-report** |  |  |  |
| Depression time 1 | .32 (.23-.40) | .15 (.09-.22) | .53 (.50-.56) |
| Depression time 2 | .33 (.23-.43) | .07 (.00-.15) | .59 (.55-.63) |
| Conduct time 1 | .39 (.30-.48) | .06 (.00-.12) | .55 (.52-.59) |
| Conduct time 2 | .36 (.31-.39) | .00 (.00-.03) | .64 (.61-.68) |
| Hyperactivity time 1 | .45 (.42-.48) | .00 (.00-.02) | .55 (.52-.58) |
| Hyperactivity time 2 | .39 (.35-.42) | .00 (.00-.02) | .61 (.58-.65) |
| **Parent-report** |  |  |  |
| Depression time 1 | .46 (.40-.52) | .21 (.16-.26) | .33 (.31-.35) |
| Depression time 2 | .42 (.34-.50) | .18 (.12-.24) | .40 (.37-.43) |
| Conduct time 1 | .49 (.44-.54) | .28 (.24-.33) | .22 (.21-.24) |
| Conduct time 2 | .64 (.58-.71) | .11 (.07-.16) | .25 (.23-.27) |
| Hyperactivity time 1 | .73 (.71-.75) | .00 (.00-.01) | .27 (.25-.29) |
| Hyperactivity time 2 | .77 (.75-.78) | .00 (.00-.01) | .23 (.22-.25) |

**Table S2** – Proportion of bivariate phenotypic correlations due to genetic (A), shared (C), and non-shared (E) environmental influences

|  | Depression time 1 | Conduct time 1 | Hyperactivity time 1 | Depression time 2 | Conduct time 2 | Hyperactivity time 2 |
| --- | --- | --- | --- | --- | --- | --- |
| Depression time 1 | - | .73/.26/.01 | .63/.21/.16 | .31/.67/.01 | .94/.06/.00 | .78/.12/.10 |
| Conduct  time 1 | .89/.02/.09 | - | .90/.07/.03 | .68/.32/.00 | .99/.01/.00 | .95/.03/.02 |
| Hyperactivity time 1 | .69/.02/.29 | .67/.00/.33 | - | .61/.27/.12 | 1.00/.00/.00 | .63/.02/.35 |
| Depression time 2 | .52/.46/.02 | .91/.04/.05 | .77/.04/.19 | - | .58/.26/.16 | .68/.13/.19 |
| Conduct  time 2 | 1.00/.00/.00 | .96/.01/.04 | .85/.00/.15 | .70/.00/.30 | - | .85/.06/.09 |
| Hyperactivity time 2 | .69/.08/.23 | .71/.00/.29 | .38/.00/.62 | .55/.05/.40 | .59/.00/.41 | - |

*Notes:*

Results are reported at proportions due to A/C/E

Self-report below diagonal, parent-report above diagonal

Homotypic (within-symptom) continuity highlighted in light grey; Heterotypic (across-symptom) continuity highlighted in dark gray.

**Table S3-** Independent pathways model, full results for self-report symptoms

|  | Common influences | | | | | | Specific influences | | |
| --- | --- | --- | --- | --- | --- | --- | --- | --- | --- |
|  | A_c1_ | A_c2_ | C_c1_ | C_c2_ | E_c1_ | E_c2_ | A_s_ | C_s_ | E_s_ |
| Depression time 1 | .29  (.23-.36) |  | .16  (.11-.21) |  | .02  (.01-.04) |  | .03  (.00-.09) | .00  (.00-.03) | .49  (.47-.52) |
| Conduct  time 1 | .49  (.46-.51) |  | .00  (.00-.01) |  | .06  (.04-.08) |  | .00  (.00-.01) | .00  (.00-.01) | .45  (.43-.48) |
| Hyperactivity time 1 | .24  (.21-.27) |  | .00  (.00-.01) |  | .49  (.40-.62) |  | .15  (.12-.17) | .00  (.00-.00) | .12  (.00-.21) |
| Depression time 2 | .06  (.04-.09) | .11  (.04-.16) | .09  (.05-.11) | .01  (.00-.07) | .00  (.00-.01) | .09  (.07-.12) | .14  (.08-.19) | .00  (.00-.03) | .50  (.46-.54) |
| Conduct  time 2 | .19  (.16-.22) | .15  (.08-.20) | .01  (.00-.02) | .00  (.00-.04) | .00  (.00-.01) | .15  (.11-.20) | .01  (.00-.06) | .00  (.00-.02) | .49  (.44-.52) |
| Hyperactivity time 2 | .08  (.06-.10) | .14  (.08-.22) | .00  (.00-.01) | .00  (.00-.04) | .10  (.07-.13) | .18  (.14-.28) | .11  (.03-.15) | .00  (.00-.00) | .38  (.33-.43) |

*Notes:*

A – additive genetic influences, C – shared-environmental influences, E- non-shared environmental influences. Subscript *c* denotes common influences and subscript *s* denotes time- and variable-specific, residual influences.

Non-significant paths were not dropped to prevent artificially inflating remaining paths. Only significant paths are presented in Figure 2.

All paths presented are squared for standardization: A, C and E influences on each variable add up to 1. Square root of these values should be taken to obtain variance path.

**Table S4** - Independent pathways model, full results for parent-report symptoms

|  | Common influences | | | | | | Specific influences | | |
| --- | --- | --- | --- | --- | --- | --- | --- | --- | --- |
|  | A_c1_ | A_c2_ | C_c1_ | C_c2_ | E_c1_ | E_c2_ | A_s_ | C_s_ | E_s_ |
| Depression time 1 | .15  (.12-.18) |  | .24  (.20-.29) |  | .01  (.00-.01) |  | .27  (.22-.32) | .00  (.00-.03) | .32  (.30-.35) |
| Conduct  time 1 | .48  (.44-.52) |  | .04  (.02-.06) |  | .00  (.00-.00) |  | .07  (.01-.12) | .20  (.16-.24) | .22  (.21-.24) |
| Hyperactivity time 1 | .36  (.32-.39) |  | .02  (.01-.04) |  | .35  (.31-.38) |  | .27  (.24-.29) | .00  (.00-.00) | .00  (.00-.04) |
| Depression time 2 | .05  (.04-.08) | .05  (.01-.12) | .15  (.11-.20) | .05  (.01-.11) | .00  (.00-.00) | .05  (.03-.07) | .29  (.22-.34) | .00  (.00-.05) | .36  (.33-.38) |
| Conduct  time 2 | .40  (.36-.43) | .06  (.01-.14) | .00  (.00-.01) | .13  (.03-.18) | .00  (.00-.00) | .06  (.04-.09) | .16  (.09-.22) | .00  (.00-.10) | .18  (.16-.21) |
| Hyperactivity time 2 | .29  (.25-.32) | .16  (.07-.43) | .00  (.00-.01) | .01  (.00-.01) | .09  (.07-.10) | .04  (.03-.06) | .25  (.00-.32) | .00  (.00-.01) | .17  (.15-.19) |

*Notes:*

A – additive genetic influences, C – shared-environmental influences, E- non-shared environmental influences. Subscript *c* denotes common influences and subscript *s* denotes time- and variable-specific, residual influences.

Non-significant paths were not dropped to prevent artificially inflating remaining paths. Only significant paths are presented in Figure 3.

All paths presented are squared for standardization: A, C and E influences on each variable add up to 1. Square root of these values should be taken to obtain variance path.

**Figure S1** - Independent pathways model, significant results for combined self- and parent-report symptoms

| **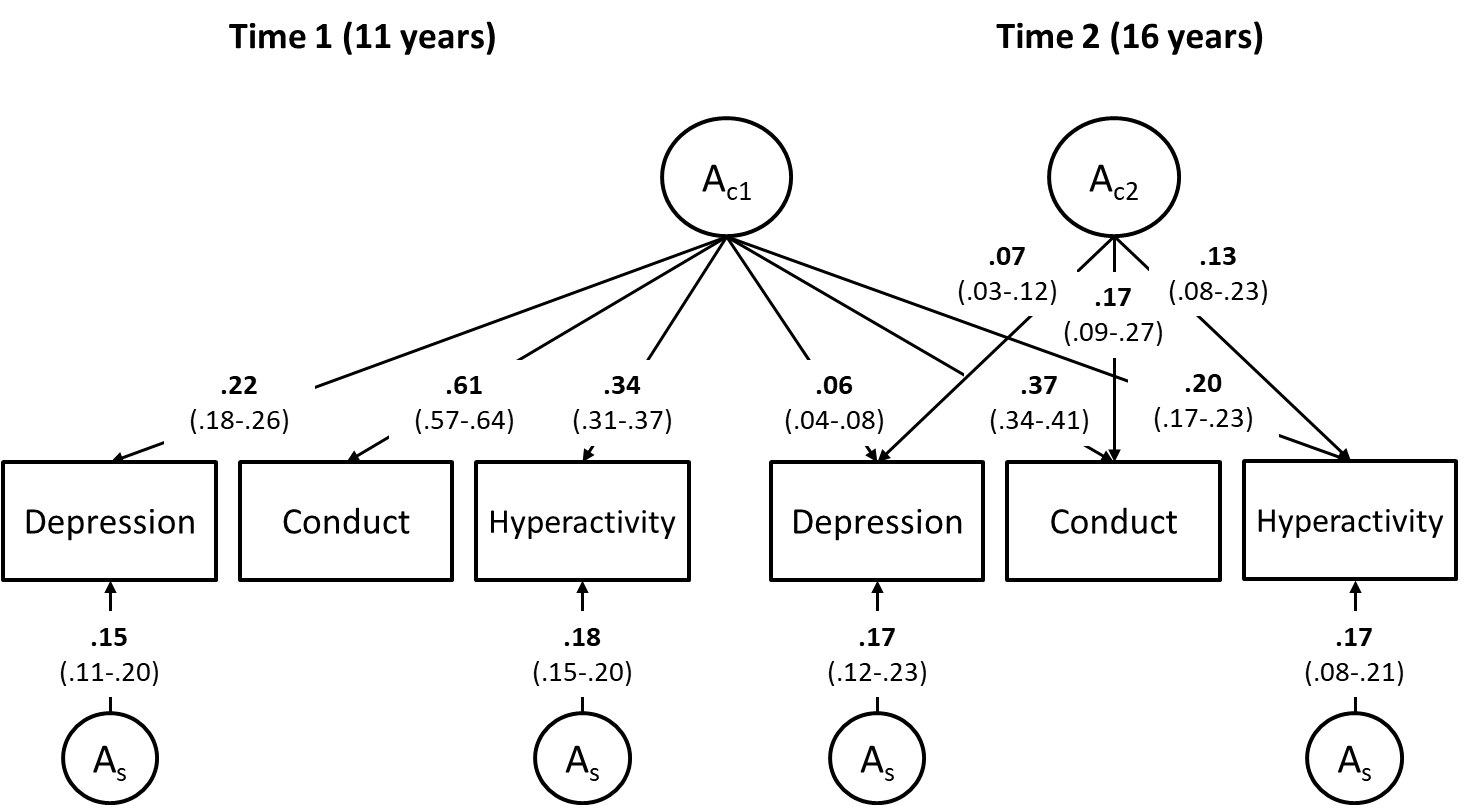** |
| --- |
| **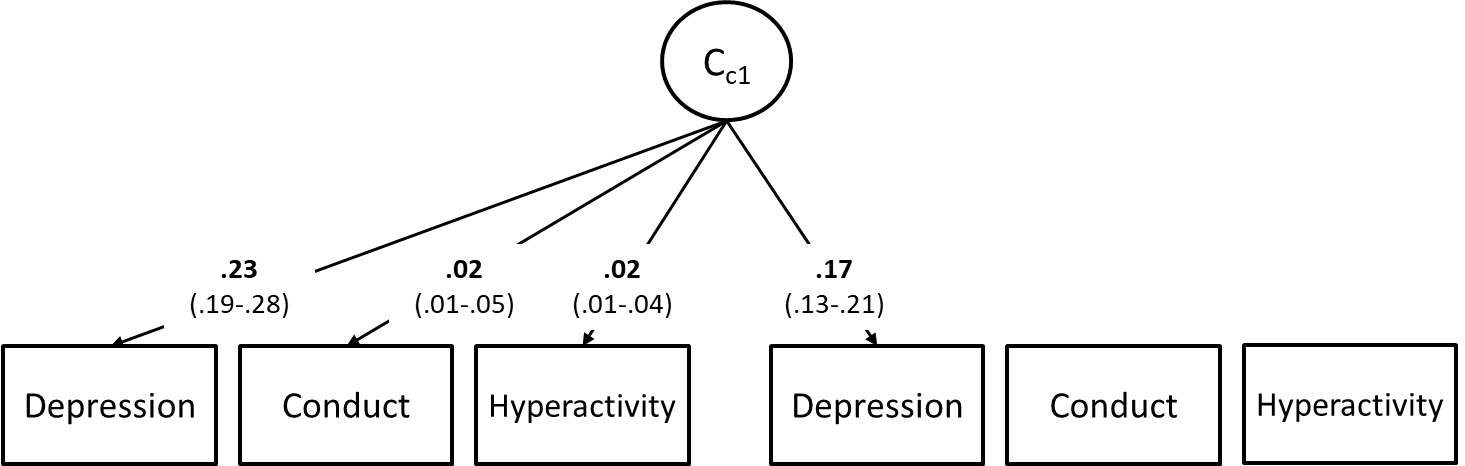** |
| **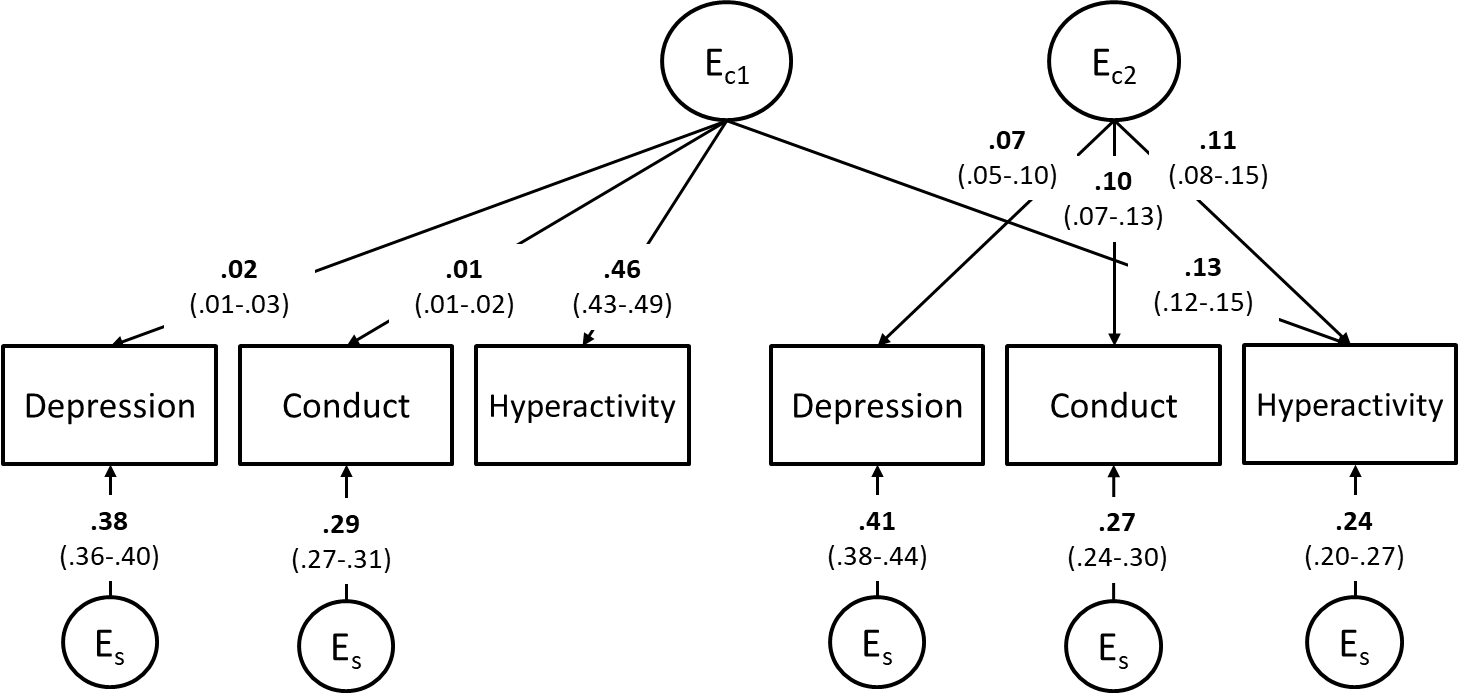** |

*Notes:* A – additive genetic influences, C – shared-environmental influences, E- non-shared environmental influences. Subscript *c* denotes common influences and subscript *s* denotes time- and variable-specific, residual influences.

Self- and parent-report variables were standardized and combined by creating an average score across both raters.

Full model presented in Figure 1 was fitted, but only results for significant paths are shown. Non-significant paths were not dropped to prevent artificially inflating remaining paths.

All paths presented are squared for standardization: A, C and E influences on each variable (including non-significant paths that are not shown) add up to 1. Square root of these values should be taken to obtain variance path.

.
